# A Hybrid Evolutionary Algorithm for Wheat Blending Problem

**DOI:** 10.1155/2014/967254

**Published:** 2014-02-20

**Authors:** Xiang Li, Mohammad Reza Bonyadi, Zbigniew Michalewicz, Luigi Barone

**Affiliations:** ^1^School of Computer Science, The University of Adelaide, Adelaide, SA 5005, Australia; ^2^Institute of Computer Science, Polish Academy of Sciences, Ulica Ordona 21, 01-237 Warsaw, Poland; ^3^Polish-Japanese Institute of Information Technology, Ulica Koszykowa 86, 02-008 Warsaw, Poland; ^4^SolveIT Software, 99 Frome Street, Adelaide, SA 5000, Australia

## Abstract

This paper presents a hybrid evolutionary algorithm to deal with the wheat blending problem. The unique constraints of this problem make many existing algorithms fail: either they do not generate acceptable results or they are not able to complete optimization within the required time. The proposed algorithm starts with a filtering process that follows predefined rules to reduce the search space. Then the linear-relaxed version of the problem is solved using a standard linear programming algorithm. The result is used in conjunction with a solution generated by a heuristic method to generate an initial solution. After that, a hybrid of an evolutionary algorithm, a heuristic method, and a linear programming solver is used to improve the quality of the solution. A local search based posttuning method is also incorporated into the algorithm. The proposed algorithm has been tested on artificial test cases and also real data from past years. Results show that the algorithm is able to find quality results in all cases and outperforms the existing method in terms of both quality and speed.

## 1. Introduction

Wheat is Australia's most important grain crop. About 80 percentage of Australia's wheat is exported. Australia is the world's fourth-largest exporter of wheat. Usually, wheat is sold to the central collection sites via truck in batches, called *loads*. When submitted, each load is weighted and sampled and the result of quality checks is given. There are 10 to 20 attributes, such as protein content and moisture, that are checked. A grade is assigned to each load according to the result of the quality check. This grade is used to deliver products (wheat) within given specifications. There are 26 grades in total; each one has its own quality requirements and price. The value of the wheat is determined by its grade (see Table 1).

For example, consider grades G1 and G2 (for simplicity, only the protein content is presented here). To be graded as G1, the protein content of wheat must be within the 11.0%–12.5% range, and for G2 the range is from 10% to 11.0%. G1 has a higher requirement on protein and has a higher price. Now let us consider three loads of wheat and how they will to be graded.

As shown in Table 2, L1 (with 11.5% of protein) is graded as G1, L2 (with 10.5% of protein) is graded as G2, and L3 (with 10.0% of protein) is graded as G2. Note that, although L1, L2 have a higher protein value than the required lower bounds, the price does not increase.

In fact, there are many cases where the quality of wheat is above the minimum requirement or cases where the wheat is just short of obtaining a higher grade. One way to improve the overall value is to *blend* the wheat.

Blending is the process of mixing wheat of different qualities. This is usually done by blending low-quality (low price) wheat with some high-quality wheat to achieve a better overall value. Blending is a vital part of the entire wheat supply chain and, as discussed below, plays a major role in generating profit.

By blending different loads, the mixture (called a *lot*) could be assigned with a new grade based on the weighted average quality result.

**Figure 1:**
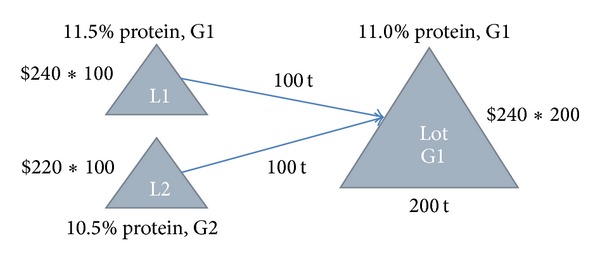


**Figure 2:**
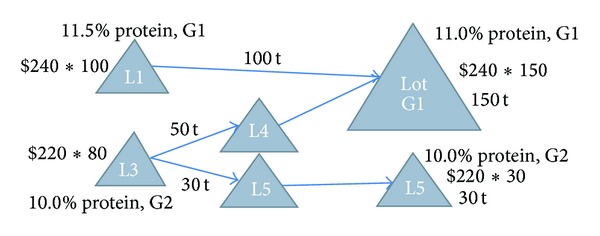


Figures 1 and 2 present two examples to illustrate the basics of blending.

In Figure 1, there are two loads, L1 and L2. L1 is 100 tonnes, with a protein percentage of 11.5% that would be graded as G1. L2 is 100 tonnes, with a protein percentage of 10.5% that would be graded as G2. The price of G1 is $240 per tonne and G2 is $220 per tonne.

Suppose that the requirement of G1 is to have at least 11.0% of protein. Clearly, L1 exceeds the protein requirement of G1 (with no additional benefit) and can be mixed with L2 to achieve a better total value. If L1 and L2 are blended together, the mixed lot will have a protein percentage of 11.0% and thus still meet the requirement of G1. This results in an increase of total value: the value before blending sums to $46,000 and the value after blending is $48,000, realising an *uplift *of $2,000.

Figure 2 represents a more complicated example. There are two loads, L1 and L3. L1 is 100 tonnes, with a protein percentage of 11.5% that would be graded as G1. L3 is 80 tonnes, with a protein percentage of 10.0% that would be graded as G2. The price of G1/G2 is still $240/220 per tonne. Since L3 has less protein than L2, in this case, the blending of L1 and L3 no longer meets the protein requirement of G1.

Instead, we can split L3 into two subloads, L4 with 50 tonnes and L5 with 30 tonnes, and then blend L1 and L4 together to form a G1 lot. In this case, the value before blending sums to $41,600 and the value after blending is $42,600, increasing profit by $1,000.

The growers in Australia do not actually do the physical blending work. However, they could sell their wheat at the blended price if a blending plan is provided. Thus, the result of a provided blending plan is directly related to their profit. In fact, for growers with hundreds of loads of wheat, the profit of blending can be easily beyond $200,000.

However, building a good blending plan is a very complex task even for experts. As an example, in Australia, the grading standard includes up to 20 attributes (protein, moisture, screening, earth, etc.) and 2 unique constraints (discussed in Section 2) to determine the grade of wheat. Moreover, one individual grower might have more than 500 loads of wheat, all with different qualities. As a result, a good blending plan often needs hours or days of work.

During the harvest season, the prices of wheat change daily or even more frequently. As the price of wheat changes, the optimal way to blend also changes. This indicates that not only the quality of the blending plan is important, but the time taken to generate that plan is also important. A good plan created after hours of work might be already outdated due to the changes of the price. There is not enough time to manually build a good blending plan every time the price changes. A tool which can generate the blending plan in a short period of time is in much demand.

This paper extends our previous work [1] where we proposed a linear programming guided hybrid evolutionary algorithm to address the wheat blending problem. The proposed algorithm is hybridized with an evolutionary algorithm, a heuristic algorithm, and a linear programming algorithm. In addition to these, a heuristic based initialization method is used to reduce the search space and a local search is also applied to fine-tune the final result. During the 2013 harvest season, the proposed algorithm helped thousands of growers build their blending plans and generate tens of millions dollars profit for the growers. In this paper, more experiment results are presented and detailed stage-by-stage performance analysis is included as well.

The rest of the paper is organized as follows. Section 2 introduces the blending problem in detail. Then Section 3 provides some background on related work for solving the underlined problem. The proposed hybrid evolutionary algorithm is described in Section 4. In Section 5, the proposed algorithm is applied to the test cases and the results of comparison with a heuristic algorithm in current use are provided. The impact of each stage is included in Section 6 and Section 7 concludes the paper.

## 2. Model of the Problem

Wheat is one of the most important agricultural commodities in Australia and is one of Australia's most valuable exports. Blending is an important stage in the whole wheat supply chain. Before milling, wheat with different levels of quality may be mixed together to balance the cost and quality. The price of wheat is based on many quality attributes and some wheat may have higher quality values than required. In these cases, high-quality wheat can be blended with low-quality wheat to balance the quality, thereby having a better overall value.

This problem can be described by the following model. Assume that *L* is the number of loads, *G* is the number of grades, *l* represents a load, and *g* represents a grade. Also, *S*_*L*, *S*_*G*, *P*_*L*_*l*_, and *P*_*G*_*g*_ represent the set of all loads, the set of all grades, the price per tonne of load *l*, and the price per tonne of grade *g* wheat, respectively. Consider that *t* is the decision variables vector defined by *t*_*l*,*g*_ which is the number of tonnes from load *l* that has been blended into grade *g* lot. The objective of this blending problem is to

(1) find  t∈RL×RG  such that maximize  ∑l∈S_L(∑g∈S_G(P_Gg−P_Ll)tl,g).

In (1), (*P*_*G*_*g*_ − *P*_*L*_*l*_) is the earned profit when load *l* is blended into a lot with grade *g*. This refers to the fact that maximizing the profit generated by blending is desirable.

Then, *M*_*l*_ is the number of tonnes of load *l* originally and (2) and (3) indicate that the total tonnes of load *l* used in blending should always be greater than or equal to 0 and less than or equal to its original tonne weight:

(2) $$\begin{array}{c} \sum_{g\in S\_G}t_{l,g}\geq 0\;\forall l, \end{array}$$

(3) $$\begin{array}{c} \sum_{g\in S\_G}t_{l,g}\leq M_{l}\;\forall l. \end{array}$$

Equations (4) and (5) are the constraints on the quality standards of each grade. *q* represents one quality attribute, for example, the protein percentage. *Q*_*q*,*l*_, *Q*_*Max*⁡_*q*,*g*_, and *Q*_*Min*⁡_*q*,*g*_ are the quality attribute *q* of load *l*, the maximum requirement of quality attribute *q* for grade *g*, and the minimum requirement of quality attribute *q* for grade *g*, respectively. The weighted average result of quality attribute *q* for the blended lot with grade *g* should always be within the min/max range:

(4) $$\begin{array}{c} \sum_{l\in S\_L}\left(Q_{q,l}t_{l,g}\right)\leq {Q\_\mathrm{Max}}_{q,g}\sum_{l\in S\_L}\left(t_{l,g}\right)\;\forall g,q, \end{array}$$

(5) $$\begin{array}{c} \sum_{l\in S\_L}\left(Q_{q,l}t_{l,g}\right)\geq {Q\_\mathrm{Min}}_{q,g}\sum_{l\in S\_L}\left(t_{l,g}\right)\;\forall g,q. \end{array}$$

Linear constraints usually cannot model real-world problems precisely. As in our problem, there are two nonlinear constraints involved which makes the problem quite unique.

Firstly, the Australian standards suggest that the weighting of wheat is precise down to the 10 kilo range. Thus 100*t*_*l*,*g*_ is required as an integer vector since it used tonnes based weighting. This hard constraint corresponds to

(6) $$\begin{array}{c} 100t_{l,g}\;\text{is integer}\;\forall l,g. \end{array}$$

There is also one more constraint which further complicates the problem. As proposed, it is possible to just take a part from load *l* to use in blending, known as a *split*. However, the total number of splits allowed for the entire blending plan is limited, and this may differ from grower to grower. This constraint is included in

(7) $$\begin{array}{c} \sum_{l\in S\_L}\left(\sum_{g\in S\_G}\left\lceil \frac{t_{l,g}}{M_{l}}\right\rceil -1\right)\leq S, \end{array}$$

where *S* is the number of splits allowed and ⌈*x*⌉ is the ceiling function which returns the smallest integer not less than *x*.

## 3. Related Work 

In this section, related algorithms for solving the general blending problem are detailed and a brief introduction to the epsilon level constraint handling is included.

### 3.1. Linear Programming

Linear programming (LP) is an optimization technique that has been designed for addressing continuous space (decision variables are continuous) optimization problems. LP requires that the objective function and constraints are all linear and LP algorithms are able to solve such optimization problem to optimality. There are many methods to solve linear programming problems such as simplex, criss-cross, and interior point methods [2].

In this blending problem, the objective function (1) and constraints (2), (3), (4), and (5) are all linear. Thus the linear relaxed version, which only considers (1) to (5), can be solved efficiently using a linear programming algorithm. There have been a few attempts to solve similar blending problems (with only linear constraints) using linear programming algorithms, especially before the early 1990s [3].

However, for this problem, (6) and (7) affect the model significantly. The result from a linear-relaxed model might break either or both of the constraints. Firstly, linear programing is operated in the continuous space; thus there is no guarantee that the result is feasible for (6). Secondly, the result might use any number of splits, which breaks (7). Both of the constraints are important for business. Constraint (6) is clearly stated in the Australian standards and (7) comes from the capacity limitation for the shared storage space. In addition, (7) is also used by the business to control hidden operational cost.

We can partially solve the problem of (6) by rounding the results. A simple half-up rounding will do the job but then the result is no longer guaranteed to satisfy all the constraints from (2) to (5). During our experiments, there are around 30% of the cases in which the result after the half-up rounding is still feasible. Some heuristic based rounding methods could increase the chance to 60%, but those methods are computationally expensive and are not the focus of this paper.

Again, we can use rounding (if the variable representation is transformed from *t*_*l*,*g*_ to (*t*_*l*,*g*_/*M*_*l*_) and capping the cases where the value is rounded up to 1) to solve the problem of (7). However, the variations needed are significant and feasibility of the solution is not guaranteed either. The result is also quite possibly a suboptimal solution, since those extra splits used usually contribute major sources of profit.

### 3.2. Mixed Integer Programming

Blending problems are also often modelled as mixed integer programming problems, especially for real-world cases [4]. Integer programming (IP) is a type of linear programming in which decision variables are integers and mixed integer linear programming (MILP) is a variety where only some of the variables are constrained to be integers. There are different methods for IP/MILP: some are exact (such as the methods which use branch and bound or cutting plane) and some are approximation methods. In the exact methods, normally the relaxed version of the problem is solved by LP and then this information is used (e.g., in branch and bound) to find optimal solutions. However, the time complexity of these methods is exponential [3].

There are many studies using exact algorithms to solve blending problems. Bilgen and Ozkarahan proposed a mixed-integer linear programming model for optimizing a wheat supply chain. The objective is to minimize the total cost for blending, loading, transportation, and storage [5]. Ashayeri et al. apply the model to the blending of chemical fertilizers [6]. Jia and Ierapetritou also use a mixed-integer linear programming model to optimize the blending of gasoline [7]. The MILP model is also used in the blending of water [8] and oil [9].

MILP could model the problem more precisely than a LP since (6) is not relaxed. However, (7) is still not solved. In addition, the execution speed is limiting the usage of exact methods in here. One grower could have up to 700 loads and it may need days of time for those exact algorithms to finish. Thus, those exact methods are not applicable for this problem. Actually, unlike academic researchers, real-world users are usually more concerned of the speed of the tool, instead of the optimality of the solution. Users might be happy to have a cup of coffee while waiting for the result, but, in general, waiting for hours is not acceptable, especially in decision support systems. As a rule of thumb, a casual user usually prefers a tool that is fast and generates a quality result, but not necessarily the optimal result.

### 3.3. Metaheuristic

Metaheuristic algorithms are also a popular choice for solving complex mixed-integer programming problems [10]. Examples include applying evolution strategy to the problem of optimal multilayer coating design [11] and to optimize chemical engineering plants [12]. Other cases include an ant colony system for optimizing electrical power distribution networks [13], a genetic algorithm to optimize the design of antenna [14], to optimize the deployment of patrol manpower [15], and to optimize exosensor distribution for smart home systems [16]. Yokota et al. proposed a genetic algorithm to solve nonlinear mixed integer programming [17] and there are many other algorithms created for solving the general MILP [18–20].

However, those algorithms are either too general or too specific for the underlying problems. To obtain the best result, real-world constraints like (7) usually need specially designed methods and intense tuning [21].

### 3.4. Evolutionary Algorithm

An evolutionary algorithm (EA) is a stochastic population-based metaheuristic that mimics biologically inspired operators such as *mutation*, *recombination,* and *selection*. In an EA, a set (known as the *population*) of initially generated solution candidates (known as *individuals*) is processed (generations: the main loop of the EA). In each generation, a subset of the individuals in the population is selected (via the selection operator, to mimic the competition between individuals). The selected individuals are then modified (via the mutation and/or recombination operator), resulting in a new set of individuals. This subset is merged into the original population and after a selection process (to mimic the “survival of the fittest” process), a new population is generated. This process is repeated until a certain termination criterion is met (such as reaching the maximum generation limit or the solution is not being improved for a long time) [22].

In many practical cases, it has been reported that hybridizing an EA with other methods is effective [23]. There are many ways to hybridize an EA with other methods. For example, one way would be to incorporate with other methods to create problem dependent operators [24]. Another way would be to apply another method to improve the final solutions found by the EA [25]; It is also possible to use problem specific representation [26] or to run one or more algorithms interactively [27].

### 3.5. Heuristic Algorithm

One existing tool has been used by growers to help them build the blending plan, and it uses a heuristic based algorithm. The heuristic is based on the fact that protein percentage is the main attribute to differentiate grades. Thus the algorithm tries to find a load that has the best (profit/protein cost) ratio. If given a load *l* and a target lot with grade *g*, the ratio can be calculated as

(8) $$\begin{array}{c} \frac{{P\_G}_{g}-{P\_L}_{l}\;}{{Q\_\mathrm{Min}}_{\text{protein},g}-Q_{\text{protein},l}}, \end{array}$$

where *P*_*G*_*g*_ is the unit price of grade *g* wheat, *P*_*L*_*l*_ is the unit price of the load *l*, *Q*_*Min*⁡_protein,*g*_ is the minimum protein requirement of grade *g*, and *Q*_protein,*l*_ is the protein percentage of load *l* respectively.

After that, the algorithm tries to find one or more companion loads which have better quality attributes to improve the weighted average quality. The combination of the selected load, the target lot, and the companion loads is called a *blend*. The algorithm stops if it cannot find any blend with profit.

The method used to find the companion loads is to do an exhaustive search with all the combination of 3 (or less) loads. The whole process is summarized as in Algorithm 1. The NOT_VALID method tests whether any constraint violation is introduced. The NO_PROFIT method tests whether any profit is generated.

This algorithm was a lot faster than doing the blending manually, but the generated result was often suboptimal. This tool could solve some simple problems but, for more complex cases, the user typically used the tool to generate a base solution and then tweaked it to get a better result (in fact, our proposed algorithm follows the same ways as the users. It generates an initial solution first and then tweaks it to get a better result). The users were generally happy with the tool but always seek for a better tool that could generate a quality blending plan all the time while keeping the execution time short.

### 3.6. Epsilon Level Constraint Handling

Epsilon (*ε*) level constraint handling (*ε* LCH) is a method that transforms the constrained optimization problems into unconstrained problems [28]. The transformation is done by replacing the ordinary comparison operator by the *ε* *level comparison* operator. The *ε* level comparison operator combines the constraint violation values and objective values for evaluating candidate solutions.

In short, the *ε* level comparison compares two solutions by their constraint violation values first. The solution which has a lower constraint violation value is ranked higher. However, if both the violation values are under a small threshold *ε*, then the constraint violation values are ignored, and the two solutions are only compared by their objective function values.

Suppose there are two candidate solutions *x*_1_ and *x*_2_, *f*_1_ and *f*_2_ are the objective values, and *h*_1_ and *h*_2_ are constraint violation values of *x*_1_ and *x*_2_; then the *ε* level comparison operator <_*ε*_ and ≤_*ε*_ is defined by the following:

(9) $$\begin{array}{c} x_{1}\;{<}_{\varepsilon }\;x_{2}\equiv \{\begin{array}{cc} f_{1}<f_{2} & \text{if}\;h_{1},h_{2}\leq \varepsilon \\ f_{1}<f_{2} & \text{if}{\;h}_{1}=h_{2}\\ h_{1}<h_{2} & \text{otherwise,} \end{array}\\ x_{1}\;\leq_{\varepsilon }\;x_{2}\equiv \{\begin{array}{cc} f_{1}\leq f_{2} & \text{if}\;h_{1},h_{2}\leq \varepsilon \\ f_{1}\leq f_{2} & \text{if}\;h_{1}=h_{2}\\ h_{1}\leq h_{2} & \text{otherwise.} \end{array} \end{array}$$

There are many ways to control the threshold *ε*. The formula used by the proposed algorithm is included in Section 4.

## 4. The Proposed Hybrid Evolutionary Algorithm

The proposed algorithm contains four stages: search space reduction, initialization, evolutionary loop, and local search. The working flow is shown in Algorithm 2.

In Sections 4.1 to 4.4, each of those stages is presented. Firstly, the algorithm tries to eliminate all the obvious bad choices using predefined rules. Then the algorithm solves the linear-relaxed version of the problem and uses the result as a clue to build an initial solution of the nonrelaxed version. After that, the algorithm tries to tweak the solution in an iterative fashion. In each iteration, there is an evolutionary algorithm to optimize the loads to blend, a heuristic to choose the right loads to split, and the use of a linear programming algorithm to find the optimal way to split. Final tune-up is done by a local search.

Additionally, Section 4.5 introduces a specially designed constraint handling method that is proposed into the algorithm to encourage the exploration of infeasible regions. Local search in the main loop is included in Section 4.6.

### 4.1. Search Space Reduction

In this stage, the algorithm tries to eliminate some obvious bad choices before it starts the stochastic process. This is done by a rule-based filtering process. These rules are based on advice from domain experts and experimental results. Some rules include the following.

- Never blend a load to a lot which requires at least another 2% of protein. As the protein percentage is generally from 10% to 14%, overcoming the 2% margin is too costly.
- Never blend a load that has an extra 1.5% of protein above the grade requirement, unless there are only few choices. This rule attempts to save the good quality loads for a better global result.

After this filtering process, the search space (number of possible blends) of the problem is greatly reduced.

The key of this stage is to ensure that there is no bad choice made. To do so, the thresholds of the rules are carefully chosen. Those values could almost guarantee that it does not have a negative impact on finding the optimal solution.

### 4.2. Initialization

Since the execution speed is crucial for this problem, the algorithm uses a heuristic based initialization method instead of any random initialization method. This might sacrificed the diversity of solutions but the algorithm could get a good basic solution with the least computation.

The algorithm starts with applying the simplex algorithm [3] to solve a linear-relaxed version of the problem. The linear-relaxed version is the same problem but only consider constraints (2) to (5). Then the algorithm uses the heuristic (8) to build a solution of the nonrelaxed problem, but with a threshold of 15. Only loads that have the profit-protein ratio greater than 15 are considered. After that, the algorithm extracts the common parts from both solutions and generates an initial solution based on them.

The threshold value 15 is a very high number for the profit-protein ratio. This is to ensure that the algorithm is not too greedy at the beginning. The simplex result is used to double check that and also serves as a clue for reaching the global optimal. The decisions made in this step are then fixed, not possible to modify by the latter stages.

The purpose of this stage is to generate a basic solution with no or few bad choices and further reduce the search space. Since we have chosen a very high threshold number, we can ensure that the decisions are all obvious good ones.

### 4.3. Evolutionary Loop

This is the main loop where the new solutions are generated. It contains an evolutionary algorithm to optimize the loads to blend, a heuristic to choose the right loads to split, and the use of a linear programming algorithm to find the optimal way to split. The operators used in this stage are as follows.

(i) Mutation: for a randomly selected load, change its allocation to a random lot.
(ii) Heuristic: it is to choose which load to split. For all the possible combinations of load *l* and target lot with grade *g*, this applies the 2-way tournament selection to choose *S* combinations from the top 2*S* that have the best value of
  (10) $$\begin{array}{c} \frac{{P\_G}_{g}-{P\_L}_{l}\;}{{Q\_\mathrm{Min}}_{\text{protein},g}-Q_{\text{protein},l}}M_{l}. \end{array}$$
(iii) Simplex algorithm: the selected loads in the heuristic step form a subproblem and the problem is solved with unlimited splits allowed using the simplex algorithm.

In each iteration, the algorithm tries to modify the existing solution by the mutation operator one or more times (by some probabilities). The probabilities are to ensure that the algorithm is possible to perform bigger variations. The generated new solution contains no split and is called *raw solution*. Then, the algorithm iterates over all the loads using the heuristic mentioned above and tries to find *S* good candidates to do the split. After that, the algorithm builds a linear-relaxed model with only the selected *S* loads and solves it using the simplex algorithm. The generated solution is called *split solution* and always satisfies (7) since there are no more than *S* variables in the model.

Thus, each solution has actually two forms: raw and split form. Note that the mutation only operates on the raw form and the simplex algorithm resulting in a solution in the split form. Also, the result of the simplex algorithm might not satisfy (6). In those cases, rounding is applied.

### 4.4. Local Search

Random modification is usually very inefficient when the result is close to the optimal point. It needs to be really lucky to find any improvement and it is often much more time consuming than doing an exhaustive search. Thus, a local search method is applied at the end to fine-tune the result. It tries to finds all possible combinations that could give an increase of profit. The procedure is as follows.

- For all possible combinations of load *l* and target lot with grade *g*, apply the one which gives the most profit until there are no combinations that could generate any profit.

### 4.5. Constraint Handling

As a highly constrained problem, the search space of this problem is generally separated by the constraints into many isolated feasible regions. The simplex result from the initialization stage is used here to guide the search jumping out of a single feasible region. The idea is to depenalise any blend that also can be found in the simplex result. Such blend might be a bad move by itself but is also possiblely a vital part of a bigger profitable blend.

More detailedly, if any blend violates any of the constraints (2) to (5) and the same blend can be found in the linear-relaxed result, its constraint violation value is reduced. The formula used is

(11) $$\begin{array}{c} c_{\text{new}}=\{\begin{array}{cc} 0.5c & \text{if}\;\left(\frac{S_{L}-S}{S}\right)\geq 0.5\\ \left(\frac{S_{L}-S}{S}\right)c & \text{if}\;0.5\geq \left(\frac{S_{L}-S}{S}\right)\geq 0.1\\ 0.1c & \text{otherwise}, \end{array} \end{array}$$

where *c* is the original constraint violation value, *c*_new_ is the reduced constraint violation value, *S* is the number of splits allowed, and *S*_*L*_ is the number of splits used by the simplex result, respectively.

Also, in this algorithm, solutions are compared using the *ε* level comparison operators. The value of *ε* is set according to the following equations:

(12) $$\begin{array}{c} \varepsilon_{0}=h\left(x_{0}\right),\\ \varepsilon_{t}=\{\begin{array}{cc} \varepsilon_{0}\left(1-\frac{1.5t}{T\_I}\right) & \text{if}\;0<1.5t<T\_I\\ 0 & \text{if}\;t\geq T\_I, \end{array} \end{array}$$

where *ε*_0_ is the initial *ε* value, *h*(*x*_0_) is the constraint violation value for the best solution in the initialization step, *ε*_*t*_ is the *ε* value at iteration *t*, and *T*_*I* is the iterations limit. This formula suggests that the methods will be focusing on finding feasible solutions when 1.5*t* ≥ *T*_*I*.

### 4.6. Local Search in the Evolutionary Loop

Within the main evolutionary loop, the generated solution also gets a chance to perform a single local search step and is used to speed up the convergence. Many different quality solutions are generated during the evolutionary loop and they are all good starting points for the local search. The algorithm only performs the local search by a single step. This is to ensure that the result is not suffering from premature convergence significantly.

### 4.7. Summary

The complete steps are shown in Algorithm 3.

The parameters are

- *n*: the number of offspring;
- *p*_*m*_: the probability of applying additional mutation;
- *p*_*l*_: the probability of applying one local search step within the evolutionary loop.

And the termination conditions are defined as

1. no improvement after *T*_*I* iterations;
2. total number of evaluations is over *T*_*E*.

## 5. Experimental Results

In this section, the proposed algorithm is applied to 20 selected real-world and 73 artificial test cases. All real-world test cases are created using the data from past years and should cover the most typical scenarios. The proposed algorithm is compared with the existing heuristic based algorithm here and the results were averaged over 20 runs for each test case.

### 5.1. Parameters Setting

The proposed algorithm has been implemented as a web service, running on distributed servers. To improve convergence, we always set the population size to 1 and use elitism selection. The main parameters in this experiment were set as follows:

- *n* = 7,
- *p*_*m*_ = 0.6,
- *p*_*l*_ = 0.1,
- *T*_*I* = 100,
- *T*_*E* = 50,000.

The values of those parameters are selected manually. This set of parameters gives the best averaged result on the 10 real-world test cases (R1–R10, see Section 5.2).

A larger population size is also tested. It is completely applicable but there is no fundamental improvement up to the size of 4. After that, the execution time is increased significantly. In cases where the population size is more than 1, the 2-way tournament selection is used.

### 5.2. Test Cases

The 10 real-world test cases (R1–R10) are selected by domain experts, aiming to cover the most typical scenarios. The number of loads ranges from 26 to 718 in each case and is the dominant factor in the complexity. R8 is the largest case and quite possibly the most complex. R6 is a combined case (loads from two growers) to test the extreme scenario. The profit generated ranges from thousands to a quarter-million dollars. Note that test cases R1–R10 do not have any limitation on the split allowed.

The result of the proposed algorithm is compared with the heuristic based tool in current use. The benchmark here is the known best results which are optimized manually by domain experts (supported by computer tools). The experts have spent weeks of time on those cases and they believe the results are good enough to be used as the benchmark.

Test cases RS1–RS10 are the same ones as R1–R10, but with only 1 split allowed. Those cases are more constrained and are harder (slower) to optimize. Note that there is no known best result in these cases (the proposed hybrid algorithm outperforms them). Instead, we use the known best result from R1–R10 to serve as the upper bound.

The 28 artificial tests (A1–A28) are simple test cases which contains many typical pitfalls. The number of loads ranges from 3 to 7. The first 20 tests (A1–A20) do not require any split to obtain the optimal solution but the rest (A21–A28) do.

There are 45 more artificial tests (AC1–AC45) which are pair-wise combination of the real-world test cases R1–R10. Those tests are more time consuming but also have more potential to optimize. The linear-relaxed result is served as the upper bound.

### 5.3. Results

Table 3 shows the result on cases where there are an unlimited allowed number of splits. With the split limit constraint relaxed, those cases are relatively easy. The proposed algorithm found a close-to-optimal result in all cases, while the heuristic algorithm only succussed in the most simple cases. N8 is the only case where the hybrid algorithm is greater than 1% from the known-best result. In all cases, the hybrid algorithm is significantly faster.

Table 4 shows the result on cases with only 1 split allowed. In real-world cases, the split limit is normally set as 1 to 10 depending on the choice made by the user. The proposed algorithm still outperforms the heuristic algorithm in terms of both quality and speed, and the results are very close to the upper bound except for RS1. Note that for RS5, RS6, and RS8, the heuristic algorithm generates better results than the cases with an unlimited split allowed. This suggests that the heuristic algorithm can easily get stuck in local optima.

Table 5 shows the result of the artificial tests (A1–A28). The generated blending plan is required to be the same or equal-valued with the precalculated optimal result to be able to pass the test. The proposed algorithm passes all the tests while the heuristic fails on 5 cases.

Table 6 shows the result of the combination cases (AE1–AE45). Again, the proposed algorithm outperforms the heuristic algorithm. AE5, AE13, AE20, AE27, and AE44 are the only cases where the results are greater than 3% from the linear-relaxed upper bound. It also shows that the heuristic algorithm is rarely generating good solutions for large test cases (like AE26, AE28, AE33, AE36, AE37, etc.). This suggests that the heuristic algorithm might be too greedy at the beginning and cannot get out of the local optima. In contrast to this, the results from the proposed algorithm do not suffer much from a large number of loads. Additionally, the running time of the proposed algorithm grows significantly slower than the heuristic algorithm.

## 6. Performance Evaluation

The proposed hybrid algorithm consists of 4 stages: search space reduction, initialization, evolutionary loop, and final tune-up. In this section, the performance evaluation on each stage is investigated.

Tables 7 and 8 show the results if each of the functions is disabled. The value column is used to indicate the loss of quality, and the time used is used to indicate the loss of speed (less than 100 means the algorithm runs faster than the full version).

The search space reduction stage requires around 7% of the running time. However, it builds a good base for further optimizing, as in some cases the quality of the solution drops if these two stages are missing.

The initialization stage greatly reduces the processing time required. For RS7, the time required is almost doubled if initialization is missing. And for R8 and RS8, it also improves the quality of result.

The main loop contributes a huge improvement on the quality of the generated solution. For cases like R3, R4, and R9, the algorithm can still provide good solutions just using initialization and posttuning, but not for the other cases. The result for RS1 to RS10 is the same as R1 to R10, since, without the main loop, the algorithm is not able to use any split.

The constraint handling methods (linear-guided and *ε* level comparison) do not require much time but could improve the result up to 30% for nontrivial cases. This suggests that the proposed algorithm is able to escape from the local optima with the constraint handling methods.

The local search in the main loop plays a major role in improving the quality of the solution. It also requires a significant chunk of time but is worthwhile. As mentioned before, at the later stage of the optimization process, an exhaustive search is usually more efficient than a stochastic variation.

The final tune-up improves the result slightly for some cases without much execution time needed. Note that the local search in the main loop could partially replace the effect of the final tune-up since they are basically the same method. This stage is to ensure that there is no missing profit.

## 7. Conclusions and Future Work

In this paper, a hybrid evolutionary algorithm for solving the Australian wheat blending problem is proposed. The algorithm starts with a filtering process to reduce the search space. The filtering is based on predefined rules suggested by domain experts. Then the algorithm generates its initial solution by extracting the common parts from both the result from the linear-relaxed version of the problem and the result from a heuristic method. The main loop of the algorithm uses a combination of an evolutionary algorithm, a heuristic method, and the simplex algorithm to improve the solution while maintaining the feasibility of the solution. For the constraint handling part, the result from the linear-relaxed problem is used in conjunction with the epsilon level comparison. Those constraint handling methods help the algorithm explore the infeasible regions more efficiently. Final tune-up is performed by a local search method. The proposed algorithm is tested on 20 real-world test cases and 73 artificial test cases. Result shows that the proposed algorithm always finds equal or better results compared with the existing heuristic algorithm.

For further study, the parameter setting of this algorithm could be investigated. One promising way to improve the algorithm is to design an adaptive way to control the mutation probability, the local search probability, and especially the threshold used in *ε* LCH.

There are also additional functionalities requested by the growers. The growers have signed a few supply contracts before the harvest and they want to fulfil their contracts with minimum cost and maximize the profit of the rest products. Additionally, sometimes it is beneficial for the growers to buy some wheat from the other growers. Therefore, the growers also want the optimizer to generate a blending plan with the consideration of trading between multiple growers. The blending of other types of wheat is also requested.

## Figures and Tables

**Algorithm 1:**
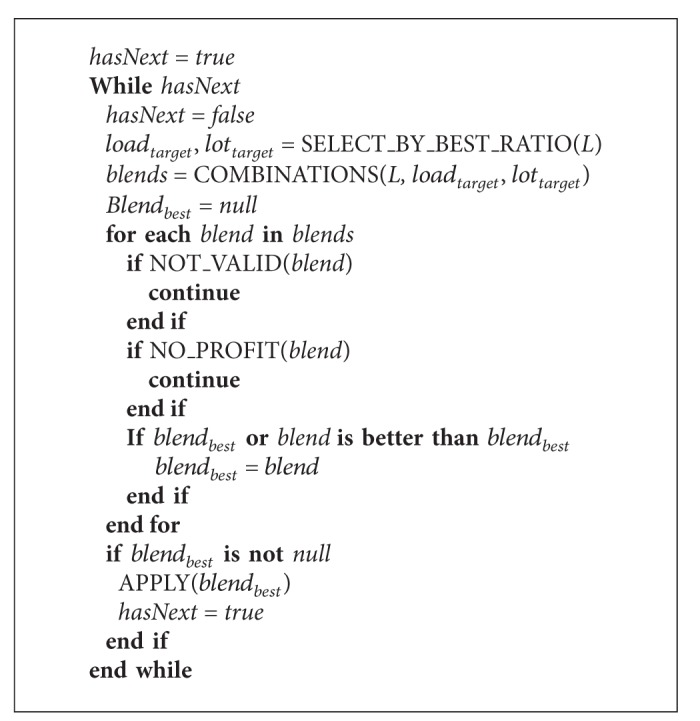
Heuristic algorithm.

**Algorithm 2:**
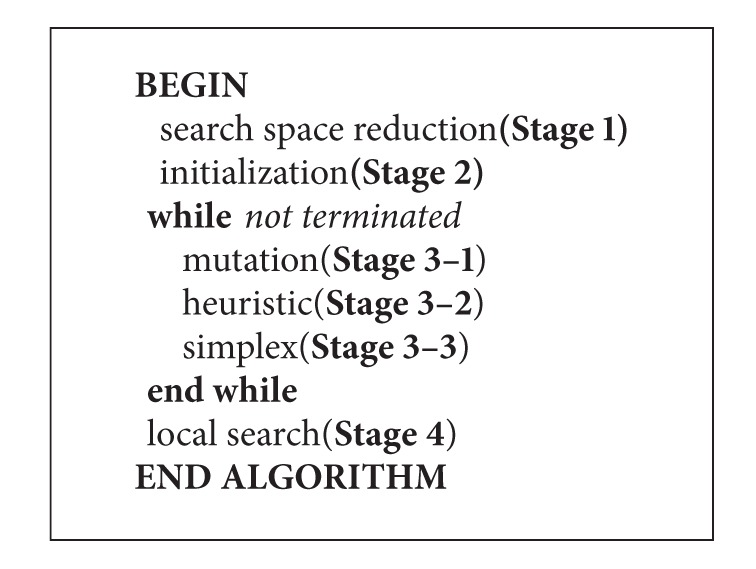


**Algorithm 3:**
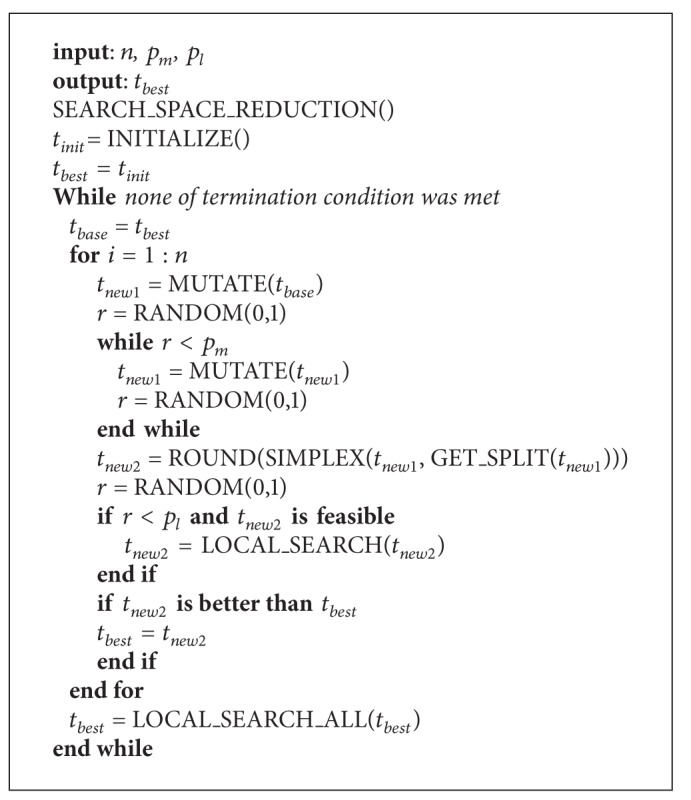


**Table 1:** 

Grade	Protein lower bound	Protein upper bound	Price per tonne
G1	11.0%	12.5%	$240
G2	10.0%	11.0%	$220

**Table 2:** 

Load	Protein	Grade	Price per tonne	Tonne
L1	11.5%	G1	$240	100
L2	10.5%	G2	$220	100
L3	10.0%	G2	$220	80

**Table 3:** Result of test cases with unlimited splits allowed.

Test case	Number of loads	Known best	Heuristic algorithm			Hybrid algorithm		
		Splits used	Percentage to known best	Splits used	Time used (seconds)	Percentage to known best	Splits used	Time used (seconds)
R1	34	3	10.3	5	15.3	0	3	1.4
R2	145	2	17.9	3	11.9	0	2	2.3
R3	26	0	0	0	1.4	0	0	1.0
R4	332	1	0	0	6.5	0	0	5.9
R5	127	2	23.7	5	22.1	0	2	2.2
R6	718	2	36.5	46	732.4	0	3	49.9
R7	129	2	15.8	2	28.9	0	3	2.2
R8	610	5	20.4	22	139.7	1.2	6	25.3
R9	49	2	2.2	7	10.1	0	2	1.6
R10	47	2	9.6	3	14.5	0	2	2.9

**Table 4:** Result of test cases with 1 split allowed.

Test case	Number of loads	Heuristic algorithm			Hybrid algorithm			
		Percentage to upper bound	Splits used	Time used (seconds)	Percentage to upper bound	Splits used	Improvement over heuristic	Time used (seconds)
RS1	34	10.6	1	11.7	6.6	1	4.4	1.4
RS2	145	18.1	1	9.4	1.8	1	19.8	2.6
RS3	26	0	0	1.4	0	0	0	1.0
RS4	332	0	0	6.5	0	0	0	5.9
RS5	127	23	1	17.2	0.1	0	29.6	3.5
RS6	718	18.4	1	572.5	0.3	1	22.2	58.6
RS7	129	15.8	1	28.4	0.5	0	18.1	2.3
RS8	610	18.4	1	85.9	1.3	0	20.9	37.8
RS9	49	2.7	0	7.8	0.5	0	2.2	1.6
RS10	47	9.8	1	12.1	0.3	1	10.4	2.9

**Table 5:** Result of test cases (A1–A28).

Test case	Number of loads	Heuristic algorithm	Hybrid algorithm
A1	4	Pass	Pass
A2	7	Pass	Pass
A3	6	Pass	Pass
A4	6	Pass	Pass
A5	3	Pass	Pass
A6	5	Pass	Pass
A7	4	Pass	Pass
A8	6	Pass	Pass
A9	3	Pass	Pass
A10	3	*Fail *	Pass
A11	3	Pass	Pass
A12	3	Pass	Pass
A13	5	Pass	Pass
A14	3	Pass	Pass
A15	5	Pass	Pass
A16	4	Pass	Pass
A17	7	Pass	Pass
A18	5	Pass	Pass
A19	4	*Fail *	Pass
A20	5	Pass	Pass
A21	7	Pass	Pass
A22	7	Pass	Pass
A23	3	*Fail *	Pass
A24	6	Pass	Pass
A25	4	*Fail *	Pass
A26	4	*Fail *	Pass
A27	3	Pass	Pass
A28	4	Pass	Pass

**Table 6:** Result of test cases (AE1–AE45).

Test case	Number of loads	Heuristic algorithm			Hybrid algorithm			
		Percentage to upper bound	Splits used	Time used (seconds)	Percentage to upper bound	Splits used	Improvement over heuristic	Time used (seconds)
AE1	179	19.7	2	21.4	2.4	2	21.49	5.5
AE2	60	4.3	1	18.0	1.8	1	2.67	3.0
AE3	366	38.0	8	192.6	1.3	5	59.36	7.5
AE4	161	22.7	1	22.3	1.4	4	27.46	5.5
AE5	752	36.0	6	912.1	7.8	12	43.92	46.4
AE6	163	12.5	6	13.5	2.2	4	11.75	4.9
AE7	644	28.5	9	491.7	1.5	8	37.85	62.5
AE8	83	4.2	1	19.1	0.7	2	3.57	2.9
AE9	81	5.2	3	18.1	1.4	1	4.00	3.7
AE10	171	26.2	1	21.9	2.5	3	32.20	6.3
AE11	477	20.5	8	57.4	0.9	8	24.70	9.1
AE12	272	15.3	4	39.0	1.6	5	16.17	4.1
AE13	863	16.5	4	738.1	4.8	7	14.01	39.2
AE14	274	19.1	5	24.7	1.0	3	22.41	5.8
AE15	755	31.1	7	438.1	1.0	9	43.67	83.1
AE16	194	20.4	2	35.6	2.1	5	23.01	3.2
AE17	192	12.5	4	20.4	1.5	4	12.54	5.5
AE18	358	28.0	5	145.8	2.3	5	35.67	7.3
AE19	153	5.3	4	16.6	1.9	5	3.66	2.8
AE20	744	31.3	9	478.6	10.6	12	30.12	77.7
AE21	155	25.2	4	15.9	1.3	4	32.05	6.3
AE22	636	36.3	6	405.6	2.2	9	53.64	48.1
AE23	75	15.1	3	13.9	0.8	4	16.94	4.4
AE24	73	17.6	2	16.7	0.3	2	20.86	3.2
AE25	459	25.9	4	89.5	1.4	7	33.05	18.0
AE26	1050	36.0	18	654.7	1.7	13	53.62	164.4
AE27	461	25.3	2	358.6	3.8	1	28.70	26.8
AE28	942	26.1	5	1796.6	0.8	10	34.26	120.9
AE29	381	8.1	12	746.7	0.6	6	8.19	12.2
AE30	379	15.4	2	510.5	0.2	4	18.00	9.6
AE31	845	32.5	9	757.2	1.7	8	45.59	89.2
AE32	256	24.6	2	289.0	0.6	5	31.83	12.8
AE33	737	52.2	6	1038.5	0.2	5	108.92	41.9
AE34	176	28.9	4	23.6	0.9	3	39.36	7.0
AE35	174	9.3	5	32.5	1.4	6	8.68	5.1
AE36	847	22.7	11	998.2	0.8	9	28.21	130.7
AE37	1328	51.6	10	1104.3	2.6	6	101.24	219.1
AE38	767	54.7	7	501.3	1.6	4	117.31	31.5
AE39	765	36.1	4	695.0	2.3	4	52.87	72.5
AE40	739	34.3	6	390.0	2.1	7	48.82	60.8
AE41	178	25.4	3	47.3	0.3	4	33.58	4.1
AE42	176	21.0	4	34.8	0.6	3	25.90	8.7
AE43	659	41.6	8	767.3	1.3	5	69.17	37.7
AE44	657	26.4	5	568.2	3.8	8	30.73	23.1
AE45	96	11.3	2	27.4	0.3	3	12.37	3.7

**Table 7:** Result of performance evaluation I.

Test case	No search space reduction		No initialization		No main loop	
	Value (percentage)	Time used (percentage)	Value (percentage)	Time used (percentage)	Value (percentage)	Time used (percentage)
R1	100	95.83	100	135.09	75.53	25.65
R2	99.13	92.86	100	178.67	80.15	32.48
R3	100	93.88	100	114.50	100	33.18
R4	100	92.24	100	120.91	100	33.12
R5	100	92.88	100	140.90	93.34	21.39
R6	98.79	92.01	100	119.67	86.92	29.90
R7	100	92.34	100	155.93	95.11	32.20
R8	98.77	93.76	99.94	154.80	72.92	22.78
R9	100	95.43	100	111.32	100	31.79
R10	100	94.53	100	135.21	95.46	26.58
RS1	100	92.22	100	112.37	75.53	25.65
RS2	97.50	92.37	100	155.02	80.15	32.48
RS3	100	95.68	100	115.96	100	33.18
RS4	100	94.95	100	143.69	100	33.12
RS5	100	93.14	100	125.32	93.34	21.39
RS6	98.69	94.32	100	112.92	86.92	29.90
RS7	100	95.30	100	195.11	95.11	32.20
RS8	96.50	94.41	99.21	115.04	72.92	22.78
RS9	100	94.87	100	118.70	100	31.79
RS10	100	93.47	100	157.74	95.46	26.58

**Table 8:** Result of performance evaluation II.

Test case	No constraint handling		No local search in loop		No final tune-up	
	Value (percentage)	Time used (percentage)	Value (percentage)	Time used (percentage)	Value (percentage)	Time used (percentage)
R1	100	97.36	97.59	82.74	100	99.87
R2	85.09	95.09	82.64	77.85	100	99.14
R3	100	96.62	100	84.63	100	98.68
R4	100	97.63	100	80.67	100	97.59
R5	73.62	95.70	83.74	77.46	100	99.33
R6	70.15	96.09	88.22	79.76	100	98.63
R7	100	94.52	95.59	86.82	100	98.20
R8	82.74	93.06	87.95	79.54	99.9	97.37
R9	100	95.97	100	87.22	100	99.73
R10	98.28	94.47	97.99	81.94	100	97.88
RS1	99.52	97.72	97.69	83.66	100	98.51
RS2	70.42	95.53	88.15	81.55	99.9	99.08
RS3	100	97.37	100	89.30	100	98.07
RS4	100	94.66	100	81.50	100	98.63
RS5	95.14	95.01	81.10	78.79	100	99.62
RS6	80.95	96.62	82.00	82.49	99.9	98.35
RS7	97.60	97.92	92.81	80.91	100	99.31
RS8	93.69	97.99	97.92	79.60	100	97.73
RS9	100	95.70	100	89.28	100	98.26
RS10	98.16	97.31	98.63	80.85	100	99.68
